# Relationship Between Type 2 Diabetes Control and Cognition in Older Adults: Findings From NHANES

**DOI:** 10.1093/geroni/igab046.2612

**Published:** 2021-12-17

**Authors:** Rozmin Jiwani, Sara Espinoza, Jing Wang, Monica Serra

**Affiliations:** 1 University of Texas Health Science Center San Antonio, San Antonio, Texas, United States; 2 University of Texas Health Science Center at San Antonio, San Antonio, Texas, United States; 3 UT Health San Antonio - Barshop Institute, San Antonio, Texas, United States

## Abstract

Cognitive health has emerged as an important public health concern for America’s aging population. Type 2 Diabetes (T2D) may be associated with an exacerbated decline in cognitive performance. This study aimed to examine the relationship between T2D control and cognitive performance in older adults (≥60 years) using the 2013-2014 National Health and Nutrition Examination Surveys. Participants who completed the following cognitive assessments were included: 1) Consortium to Establish a Registry for Alzheimer’s Disease Word List (CERAD-WL), 2) Animal Fluency (AF), 3) Digit Symbol Substitution Test (DSST) (higher scores associated with better cognition). Participants were stratified by four groups: no T2D (N=557), treated/controlled T2D (controlled; N=41), treated/uncontrolled T2D (uncontrolled; N=120), untreated T2D (N=86), based on self-reported T2D treatment, fasting plasma glucose, and hemoglobin A1c. Logistic regression was used to examine the relationship between T2D control and cognition. We observed that those with uncontrolled and untreated T2D each had ~15% lower DSST than those with no T2D (P<0.01). CERAD-WL and AF were similar across all groups. Unadjusted analyses showed that individuals with 1) lower CERAD-WL were more likely to have controlled and untreated T2D, 2) lower AF were more likely to have controlled and uncontrolled T2D, and 3) lower DSST were more likely to have uncontrolled and untreated T2D (P’s<0.05). After adjusting for significant demographics and cardiovascular risk factors, only having uncontrolled T2D was associated with lower DSST (β=-3.164, P=0.04). These data indicate the need for longitudinal studies to further explore dynamic relationship and causal pathway between T2D control and cognitive impairment.

